# Protective Effect of Ligustrazine on Lumbar Intervertebral Disc Degeneration of Rats Induced by Prolonged Upright Posture

**DOI:** 10.1155/2014/508461

**Published:** 2014-04-29

**Authors:** Qian-Qian Liang, Dao-Fang Ding, Zhi-Jie Xi, Yan Chen, Chen-Guang Li, Shu-Fen Liu, Sheng Lu, Yong-Jian Zhao, Qi Shi, Yong-Jun Wang

**Affiliations:** ^1^Institute of Spine, Shanghai University of Traditional Chinese Medicine, 725 Wan-Ping South Road, Shanghai 200032, China; ^2^Department of Orthopaedics & Traumatology, Longhua Hospital, Shanghai University of Traditional Chinese Medicine, Shanghai, China

## Abstract

Most chronic low back pain is the result of degeneration of the lumbar intervertebral disc. Ligustrazine, an alkaloid from Chuanxiong, reportedly is able to relieve pain, suppress inflammation, and treat osteoarthritis and it has the protective effect on cartilage and chondrocytes. Therefore, we asked whether ligustrazine could reduce intervertebral disc degeneration. To determine the effect of ligustrazine on disc degeneration, we applied a rat model. The intervertebral disc degeneration of the rats was induced by prolonged upright posture. We found that pretreatment with ligustrazine for 1 month recovered the structural distortion of the degenerative disc; inhibited the expression of type X collagen, matrix metalloproteinase (MMP)-13, and MMP3; upregulated type II collagen; and decreased IL-1**β**, cyclooxygenase (COX)-2, and inducible nitric oxide synthase (iNOS) expression. In conclusion, ligustrazine is a promising agent for treating lumbar intervertebral disc degeneration disease.

## 1. Introduction

Chronic low back pain, resulting from intervertebral disc degeneration [1, 2], is one of the most common and costly musculoskeletal pain syndromes of modern society [3, 4]. Degeneration of intervertebral discs (IVD) plays a central role in the pathogenesis of discogenic pain, disc herniation, and spinal instability and stenosis [5, 6]. The IVD health is influenced by the state of the adjacent vertebral endplate. Nutrient diffusion through the endplate to the IVD is critical for the maintenance of normal IVD function [7, 8]. Pathologic changes in endplate cartilage are closely related to IVD degeneration [9, 10]. Prevention and reduction of lesions in vertebral endplate are therefore a high research priority.

Chuanxiong is a dry root of the Chuanxiong plant. In ancient China this plant was usually used to treat low back pain. Ligustrazine is an alkaloid from Chuanxiong, and its chemical structure is tetramethylpyrazine. Currently it can be synthesized. Ligustrazine reportedly is able to relieve pain and suppress inflammation [11–14]. Intra-articular injection of ligustrazine can treat osteoarthritis [12]. And ligustrazine has the protective effect on cartilage and chondrocytes [15]. However, little information is available about the effect of ligustrazine on low back pain and lumbar intervertebral disc degeneration.

In this study, we aimed to examine the effect of ligustrazine on intervertebral disc degeneration. In vivo, a rat model of lumbar spine disc degeneration [16] was used to determine the protective effect of ligustrazine on disc degeneration.

## 2. Materials and Methods

### 2.1. Drug Preparation

**Figure 1:**
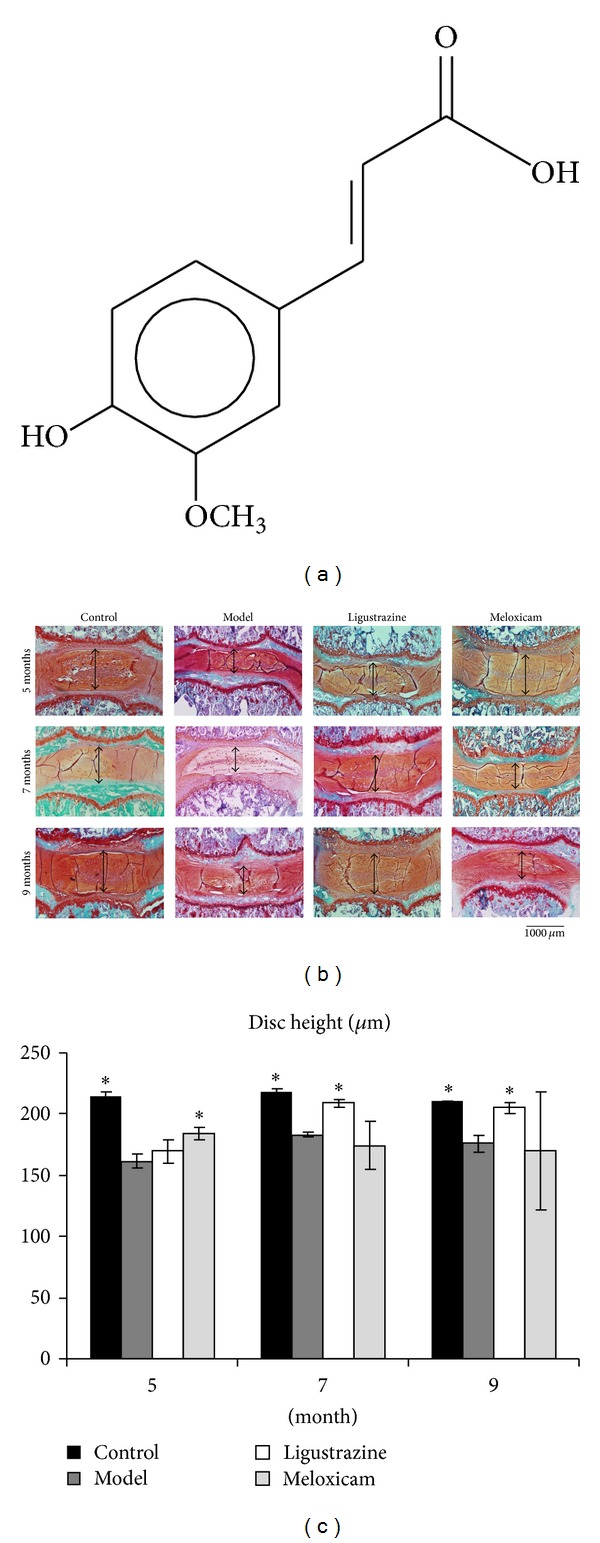
Chemical structure of ligustrazine and photomicrographs of IVDs stained by safranin-O/fast green. (a) Chemical structure of ligustrazine (tetramethylpyrazine). (b)-(c) Upright posture reduced the heights of IVDs. However, the heights of IVDs increased when ligustrazine pretreatment began seven or nine months after surgery, while meloxicam increased the heights of IVDs when pretreatment started five months after surgery and had no significant effect when pretreatment commenced seven and nine month after surgery. The arrow indicates the quantitative measurements of IVD heights; the bar equals 1000 *μ*m. Each value represents the mean ± S.D of 6 sections. *P < 0.05 compared to model group in the same month.

Ligustrazine, another name is tetramethylpyrazine (Figure 1(a)), belongs to pyrazine alkaloids [17]. The ligustrazine hydrochloride in our study was purchased from Nanning Maple Leaf Pharmaceutical Co., Ltd (Nanning, Guangxi province, China (lot number: 051125)). The storage location of the specimen used in our study is the institute of Spine, Shanghai University of Traditional Chinese Medicine, China. We used meloxicam, a nonsteroidal anti-inflammatory drug (NSAID), as a positive control drug in our experiments because it is widely used as a pain reliever in clinical treatment of intervertebral disc degeneration disease [18, 19]. We purchased it from Boehringer Ingelheim Shanghai Pharmaceutical Co., Ltd. (national medical license number: H20020217, 7.5 mg/pill).

### 2.2. Animal Model

All experiments were approved by the Animal Ethics Committee of Shanghai University of Traditional Chinese Medicine. One hundred and twenty one-month-old male Sprague-Dawley rats (Shanghai Laboratory Animal Center of Chinese Academy of Science) were randomized into the following 12 groups (*n* = 10 for each group): 5-, 7-, and 9-month control group, 5-, 7, and 9-month model group, 5-, 7-, and 9-month ligustrazine group, and 5-, 7-, and 9-month meloxicam group. Rats in the “control” groups underwent no treatment and were raised in ordinary cages.

Both forelimbs of rats in the “model,” “ligustrazine,” and “meloxicam” groups were amputated from shoulder joints via an anterior approach. And they were forced to keep upright posture in custom-made cages for 5, 7, or 9 months [16]. Four, six, and eight months after the surgery, ligustrazine and meloxicam groups were treated intraperitoneally with ligustrazine hydrochloride injections (16 mL/kg·d, 10 mL sterile saline: 40 mg ligustrazine hydrochloride) or meloxicam (0.125 mg/kg·d) for 30 days. The rats from five, seven, and nine month groups were killed at the fifth, seventh, and ninth month after surgery, respectively, and their lumbar spines were harvested for analysis.

### 2.3. Histological Evaluation

The samples containing the intervertebral discs and adjacent vertebral endplates from L4-L5 were fixed (4% paraformaldehyde; 24 hours), decalcified (20% ethylenediaminetetraacetic acid; pH 7.4; 21 days), dehydrated (gradient ethanol), cleared (dimethylbenzene), and then embedded in olefin. At least 4 consecutive 7 *μ*m sections were obtained from the sagittal planes and stained (safranin-O/fast green). Sections were examined (photomicroscope set: Olympus DP71; Olympus, Tokyo), and morphometric study was performed (image autoanalysis system: CMIAS-99B; Okolab, Milan, Italy).

### 2.4. Immunostaining for Type II Collagen and IL-1*β*

Tissue sections were dewaxed, cleared, and rehydrated. After incubating in 3% H_2_O_2_ to block endogenous peroxidase activity for 15 minutes and digestion with protease K for 10 minutes, the sections were incubated with 5% bovine serum albumin (BSA) solution in PBS for 1 hour. The sections were then incubated with rabbit polyclonal antibody to type II collagen (Abcam; Cambridge, UK; 1 : 100 dilution) and IL-1*β* (Abcam; 1 : 100 dilution) at 4°C for 12 hours. After thorough wash, the sections were incubated with biotinylated goat anti-rabbit IgG at 4°C for 60 minutes and then with Streptavidin-HRP at 37°C for 10 minutes. Color reaction were elicited by 3,3′-diaminobenzidine (DAB) solution. The sections were counterstained with hematoxylin and mounted. Sections were examined using a photomicroscope set (Olympus DP71).

### 2.5. Total RNA Isolation from Tissue

In accordance with the manufacturer's protocol (Sigma, St. Louis, MO), TRIzol reagent (1 mL) was used to isolate total RNA from intervertebral disc samples. The isolated RNA was stored at −80°C.

### 2.6. Real-Time PCR Analysis

In accordance with the manufacturer's protocol (advantage RT-for-PCR kit; Takara; Biotechnology Co., Ltd.; Dalian, Liaoning, China), total RNA (1 *μ*g) was reverse-transcribed to synthesize cDNA. Quantitative real-time PCR amplifications were carried out in Rotor-Gene real-time DNA amplification system (Corbett Research; Sydney, Australia) using 1 *μ*L of cDNA and SYBR Green (Bio-Bad; Hercules, CA) in accordance with the manufacturer's protocol. The primers for Col2*α*1, Col10*α*1, MMP3, MMP13, IL-1*β*, COX2, iNOS, and *β*-actin were designed by TaKaRa Biotechnology Co. Ltd. (see Table 1). Gene expression was normalized to *β*-actin and expressed as fold change relative to the expression values in the control groups. PCR products were subjected to melting curve analysis, and the data were quantified using Rotor-Gene 6.0 analysis software.

### 2.7. Statistical Analysis

Data are expressed as means ± standard deviation. Statistical analysis was performed (SPSS 10.0; Chicago, IL). One-way ANOVA test was used, followed by Dunnett's test for multiple comparisons. A *P* value of <0.05 was taken as statistically significant.

## 3. Results

### 3.1. Histological Observation of the Effect of Ligustrazine on IVD Degeneration

**Figure 2:**
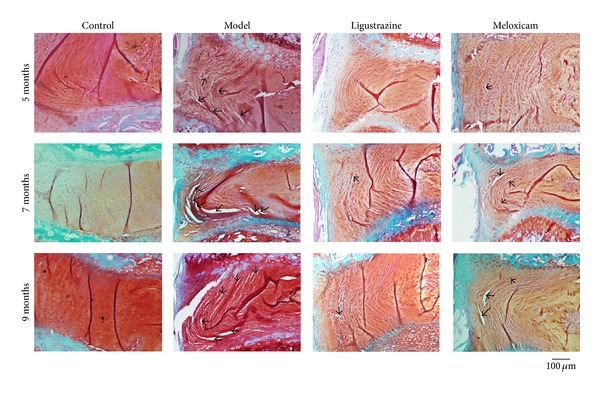
Photomicrographs of IVDs (annulus fibrosus) stained by safranin-O/fast green staining. In the surgery group the laminar structures of the annulus fibrosus were fissures at all time points. Ligustrazine almost completely reversed this disorganization at all time points, which has better effect than meloxicam. The arrow indicates the fissures of annulus fibrosus. Bar equals 100 *μ*m.

To investigate the effect of ligustrazine on IVD degeneration, we applied an upright posture induced IVD degeneration rat model [16]. We found that in control groups, the intervertebral disc between L4-L5 and adjacent endplates appeared well-organized in annulus fibrosus, endplate, and nucleus pulposus (see Figures 1 and 2). Prolonged upright posture decreased the disc height at all time points. Meloxicam pretreatment could increase the disc height at the fifth month after surgery; it was unsuccessful when pretreatment was postponed to the seventh and ninth month after surgery. The pretreatment with ligustrazine for one month could increase the disc height of upright rats when started seven or nine months after surgery but had no effect when started five months after surgery (see Figure 1).

Prolonged upright posture led to disorganization and fracture of the lamellar architecture of both inner and outer parts of the annulus fibrosus at all time points. When started five or seven months after surgery, ligustrazine pretreatment almost completely reversed the fissures of the inner part of annulus fibrosus subjected to upright posture. When started nine months after surgery, it lessened fissures at all layers of annulus fibrosus—better than the effect of meloxicam (see Figure 2). These results suggested that ligustrazine is an effective treatment on lumbar disc degeneration induced by upright posture.

### 3.2. Immunohistochemical Findings of the Effect of Ligustrazine on Type II Collagen Protein Expression at Nucleus Pulposus

**Figure 3:**
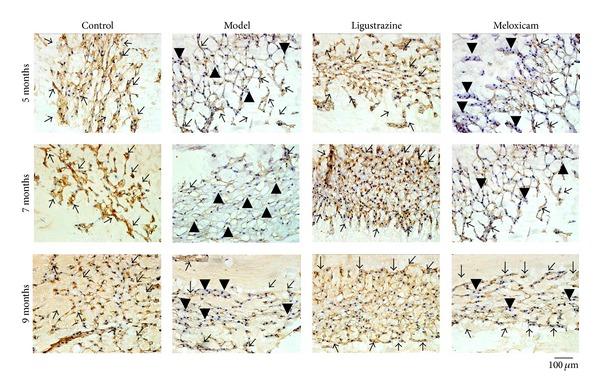
Immunohistochemical assessments of the protein level of type II collagen. Positive staining of type II collagen declined in the nucleus pulposus at all time points after the surgery. Ligustrazine treatment completely reversed the decreased trend of type II collagen at the nucleus pulposus, while meloxicam has no obvious increasing effect. The arrow indicates the positive staining cells; the arrow head indicates the negative staining cells. Bar equals 100 *μ*m.

It was reported that type II collagen is an important component of extracellular matrix of IVDs and plays an essential biomechanical function in the normal disc [18–22]. Thus, we examined type II collagen protein expression by immunohistochemical staining. We found strong immunoreactivity for type II collagen in the nucleus pulposus in control samples. Much weaker immunostaining for type II collagen was observed at any time points in the model group. The density of the type II collagen-positive staining of the ligustrazine group increased dramatically compared to the model group. Meloxicam had no effect on the protein expression of type II collagen (see Figure 3).

### 3.3. Ligustrazine Inhibited the Upright Posture-Induced Downregulation of Col2*α*1 and Upregulation of Col10*α*1, MMP13, and MMP3 mRNA Expression

**Figure 4:**
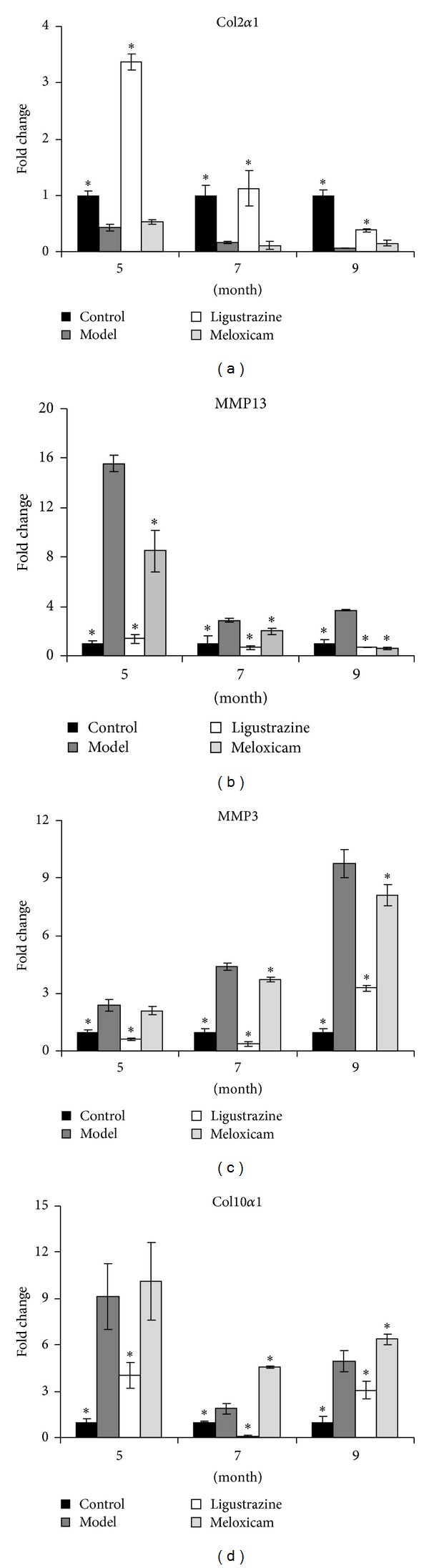
The mRNA expression of Col2*α*1, Col10*α*1, MMP3, and MMP13. (a) In the operated rats, the mRNA expression of Col2*α*1 decreases significantly at all time points but upregulated significantly after pretreatment with ligustrazine, while meloxicam has no effect on Col2*α*1 expression. (b)-(c) MMP3 and MMP13 mRNA expression increased significantly at all time points in model groups, but ligustrazine and meloxicam treatment decreased gene expression. (d) Col10*α*1 mRNA expression increased significantly at all time points in model groups and ligustrazine treatment decreased gene expression significantly at all time points, while meloxicam has no deducing role on its expression. The columns represent the mean ± SD of three independent experiments. *P < 0.05 compared to model group in the same month.

The protein expression level of type II collagen was affected by its synthesis and catabolism. Therefore, we tested the mRNA expression of Col2*α*1 and its degradation enzymes MMP13 and MMP3. We found that the mRNA expression of Col2*α*1 was significantly reduced by upright posture at all three time points and that meloxicam pretreatment had no effect on Col2*α*1 mRNA expression after surgery. In contrast, the decrease of Col2*α*1 mRNA expression was completely recovered by pretreatment with ligustrazine at all time points (see Figure 4(a)). It indicates that ligustrazine affects type II collagen synthesis directly, while meloxicam has no effect on its synthesis.

On the other hand, upright posture increased mRNA expression of MMP13 and MMP3 at all time points, but both meloxicam and ligustrazine could significantly reverse the upregulation of MMP13 and MMP3 mRNA expression induced by upright posture (see Figures 4(b) and 4(c)). These data suggested that meloxicam and ligustrazine had similar effects when it comes to reducing the degradation of enzyme expression.

It is known that type X collagen plays an important role in endochondral ossification and matrix calcification of endplate cartilage, and that its increase indicates the terminal stage of intervertebral disc degeneration [23–27]. Thus, we investigated the mRNA expression of Col10*α*1, and we found that upright posture increased the mRNA expression of Col10*α*1 at all time points and meloxicam had no effect on Col10*α*1 expression. However, ligustrazine could significantly reverse the upregulation of Col10*α*1 mRNA expression induced by upright posture at all time points (Figure 4(d)).

### 3.4. Effect of Ligustrazine on IL-1*β* Protein Distribution

**Figure 5:**
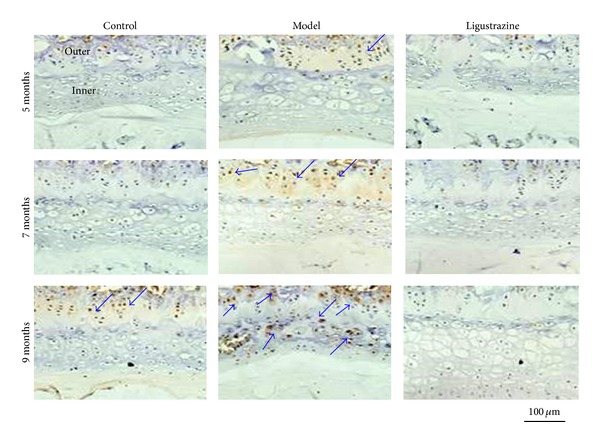
Immunohistochemical assessments of the protein level of IL-1*β*. Positive staining of IL-1*β* increased in the cartilage endplate at all time points after the surgery. Ligustrazine treatment completely blocked the increase of IL-1*β* at the endplate. Arrows indicate the positive staining cells; outer, the outer layer of endplate; inner, the inner layer of endplate. Bar equals 100 *μ*m.

It was reported that inflammatory cytokines involve the imbalance of synthesis and catabolism of extracellular matrix [28, 29]. Our previous data showed that ligustrazine could improve type II collagen synthesis and inhibit its degradation. We asked ourselves whether ligustrazine has anti-inflammatory effects. We examined the effect of ligustrazine on IL-1*β* expression and found that in the control groups IL-1*β* positive staining was very weak and limited at the outer layer of the endplate at all time points. However, in the model groups, there were more IL-1*β* positive staining cells in the outmost layer of the endplate five and seven months after surgery and distributed to both outer and inner layer of endplate nine months after surgery. Interestingly, immunostaining for this inflammatory mediator was very weak in the ligustrazine groups at both layers of endplate at all time points (see Figure 5).

### 3.5. Ligustrazine Blocked Upright Posture Induced Upregulation of IL-1*β*, iNOS, and COX2 mRNA Expression

**Figure 6:**
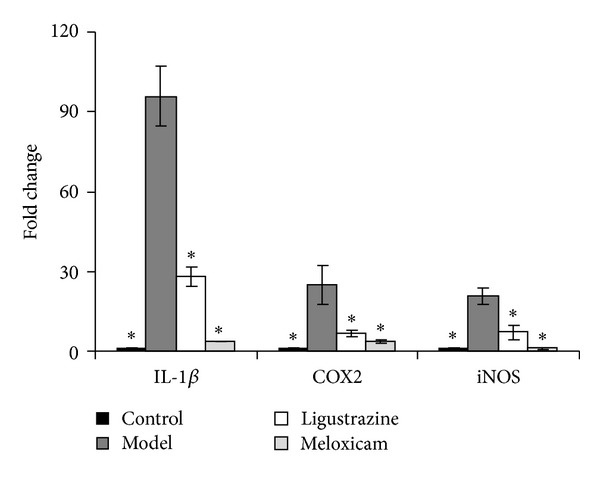
The mRNA expression of IL-1*β*, iNOS, and COX2 of intervertebral discs from the 7-month groups. Compared with control groups, upright posture increased mRNA expression of IL-1*β*, iNOS, and COX2 significantly, but ligustrazine and meloxicam pretreatment downregulated those gene expression. The columns represent the mean ± SD of three independent experiments. *P < 0.05 compared to model group.

Finally, we tested the effect of ligustrazine and meloxicam on several inflammation related factors of intervertebral discs. We tested the mRNA expression of IL-1*β*, iNOS, and COX2 of four 7-month groups and found that long term upright posture increased IL-1*β*, iNOS, and COX2 mRNA expression. However, this kind of effect was reversed by both ligustrazine and meloxicam pretreatment (Figure 6). Those data revealed that ligustrazine has similar anti-inflammatory effects as meloxicam.

## 4. Discussion

In this study, we aimed to determine whether ligustrazine could prevent intervertebral disc degeneration. We therefore carried out an in vivo study to illustrate the effect of ligustrazine in lumbar intervertebral disc degeneration induced by upright posture. We found that ligustrazine could increase the disc height and improve the morphology of the IVDs back to normal, upregulated type II collagen expression, and downregulated degradation enzymes (type X collagen and inflammatory cytokines expression). These results suggested that ligustrazine is an effective substance against lumbar disc degeneration induced by upright posture.

In our study, we applied meloxicam as positive control, because it was reported that ligustrazine reduces inflammatory response following permanent focal cerebral ischemia [30] and traumatic spinal cord [31], regulates inflammation mediators of cardiovascular disease [32], and inhibits TNF*α*, IL-1*β*, iNOS, NF-*κ*B p65, COX-2, and IL-8 expression [33, 34]. We sought to compare the different therapeutic effects between ligustrazine and meloxicam. Our study indicates that both meloxicam and ligustrazine could prevent inflammatory mediators' expression in intervertebral discs that meloxicam is better than ligustrazine and that ligustrazine has an anti-inflammation mechanism different from meloxicam. Previous reports revealed that ligustrazine could decrease IL-8 expression by blocking ERK1/2 and p38 phosphorylation [35] and reduce TNF-*α*, iNOS, NF-*κ*B p65, and COX-2 expression by increasing PPAR-*γ* signaling [34]. It was also reported that IL-1-induced ERK phosphorylation is dependent on intracellular Ca^2+^ signal [36]. Ligustrazine is a calcium antagonist [18, 37, 38] which could effectively reduce the concentration of calcium. Its calcium antagonist feature might explain ligustrazine's anti-inflammatory effect on chondrocytes.

In addition, meloxicam could only affect the expression of the IL-1*β* signaling target gene MMP13 and MMP3 but not Col2*α*1 and Col10*α*1. Moreover, it did not show better effect on intervertebral disc structure while ligustrazine not only reduced degradation enzymes expression but also increased Col2*α*1 and reduced Col10*α*1 expression. These results suggested that the anti-inflammation effect alone is not enough to counter IVD degeneration diseases; rather, ligustrazine has anti-inflammatory and extracellular matrix synthesis improving effects.

Pretreatment with ligustrazine inhibited Col10*α*1 mRNA expression at all time points. This effect suggested that ligustrazine prevents endochondral ossification and matrix calcification at endplate cartilage. It was reported that types X collagen could interact with cell surface-expressed annexin V; stimulated annexin V-mediated Ca^2+^ influx, leading to an increased intracellular Ca^2+^ concentration; and eventually increased alkaline phosphatase activity and mineralization of growth plate chondrocytes [29]. Exogenous extracellular calcium could induce the synthesis of type X collagen in chondrocytes [39]. Ligustrazine, a calcium antagonist, blocks not only the entry of extracellular calcium through calcium channels but also the release of intracellular stored calcium [18, 37, 38]. This suggested that the inhibitory effect of ligustrazine on type X collagen expression might be due to its calcium channel blocker characteristic.

Besides that, ligustrazine could regulate capillary permeability and microcirculatory perfusion [40, 41], has antioxidation effect [41, 42] and the ability to penetrate the blood-brain barrier [43]. Whether all those abilities of ligustrazine are involved in its protective effect on IVD degeneration deserves further research.

For the first time, ligustrazine was demonstrated to be effective in the lumbar intervertebral disc degeneration induced by prolonged upright posture. The findings in this study suggest that ligustrazine is a promising drug candidate for the therapy of IVD degeneration related diseases.

## Figures and Tables

**Table 1:** Sequences of primers used in the real-time RT-PCR.

Genes	Forward primer	Reverse primer
Col2*α*1 (112 bp)	F: 5′-TCCTAAGGGTGCCAATGGTGA-3′	R: 5′-GGACCAACTTTGCCTTGAGGAC-3′
MMP 13 (142 bp)	5′-CCCTGGAGCCCTGATGTTT-3′	5′-CTCTGGTGTTTTGGGGTGCT-3′
Col10*α*1 (143 bp)	5′-TTCACAAAGAGCGGACAGAGA-3′	5′-TCAAATGGGATGGGAGCA-3′
*β*-actin (150 bp)	5′-GGAGATTACTGCCCTGGCTCCTA-3′	5′-GACTCATCGTACTCCTGCTTGCTG-3′
iNOS (101 bp)	5′-CTCACTGTGGCTGTGGTCACCTA-3′	5′-GGGTCTTCGGGCTTCAGGTTA-3′
IL-1*β* (120 bp)	F: 5′-AGGTCGTCATC ATCCCACGAG-3′	R: 5′-GCTGTGGCAG CTACCTATGTCTTG-3′
COX-2 (145 bp)	F: 5′-GGAGCATCCTGAGTGGGATGA-3′	R: 5′-AAGCAGGTCTGGGTCGAACTTG-3′
MMP3 (104 bp)	F: 5′-TGGACCAGGG ACCAATGGA-3′	R: 5′-GGCCAAGTTCA TGAGCAGCA-3′

MMP-13: matrix metalloproteinase 13; IL-1*β*: interleukin 1*β*; COX-2: cyclooxygenase 2; MMP-3: matrix metalloproteinase 3; iNOS: inducible nitric oxide synthase 2.
